# Role of Type IV Pili in Predation by *Bdellovibrio bacteriovorus*

**DOI:** 10.1371/journal.pone.0113404

**Published:** 2014-11-19

**Authors:** Ryan M. Chanyi, Susan F. Koval

**Affiliations:** Department of Microbiology and Immunology, University of Western Ontario, London, Ontario, Canada; Institut Pasteur, France

## Abstract

*Bdellovibrio bacteriovorus*, as an obligate predator of Gram-negative bacteria, requires contact with the surface of a prey cell in order to initiate the life cycle. After attachment, the predator penetrates the prey cell outer membrane and enters the periplasmic space. Attack phase cells of *B. bacteriovorus* have polar Type IV pili that are required for predation. In other bacteria, these pili have the ability to extend and retract via the PilT protein. *B. bacteriovorus* has two *pilT* genes, *pilT1* and *pilT2*, that have been implicated in the invasion process. Markerless in-frame deletion mutants were constructed in a prey-independent mutant to assess the role of PilT1 and PilT2 in the life cycle. When predation was assessed using liquid cocultures, all mutants produced bdelloplasts of *Escherichia coli*. These results demonstrated that PilT1 and PilT2 are not required for invasion of prey cells. Predation of the mutants on biofilms of *E. coli* was also assessed. Wild type *B. bacteriovorus* 109JA and the *pilT1* mutant decreased the mass of the biofilm to 35.4% and 27.9% respectively. The *pilT1pilT2* mutant was able to prey on the biofilm, albeit less efficiently with 50.2% of the biofilm remaining. The *pilT2* mutant was unable to disrupt the biofilm, leaving 92.5% of the original biofilm after predation. The lack of PilT2 function may impede the ability of *B. bacteriovorus* to move in the extracellular polymeric matrix and find a prey cell. The role of Type IV pili in the life cycle of *B. bacteriovorus* is thus for initial recognition of and attachment to a prey cell in liquid cocultures, and possibly for movement within the matrix of a biofilm.

## Introduction

*Bdellovibrio bacteriovorus* is a Gram-negative obligate predator of other Gram-negative bacteria. The cells are small, vibroid in shape and highly motile via a single polar sheathed flagellum. Their life cycle consist of two stages, a motile attack phase and an intraperiplasmic growth phase. During the attack phase the cells will reversibly attach to potential prey cells for a short recognition period. If deemed suitable, *B. bacteriovorus* will irreversibly attach to the prey cell and begin to secrete hydrolytic enzymes to create a pore in the outer membrane and the peptidoglycan of the prey [1]. *B. bacteriovorus* will squeeze through the pore into the periplasmic space. The pore is resealed and an osmotically stable ‘bdelloplast’ is formed. This signifies the end of the attack phase and the beginning of the growth phase.

Evans *et al.* [2] showed the presence of polar pili on *B. bacteriovorus* and that the disruption of the *pilA* gene abolished predation. Mahmoud and Koval [3] showed that these fibers were in fact Type IV pili (TFP) and that *B. bacteriovorus* in a coculture containing anti-PilA antibody was not able to prey. These results indicate that a direct interaction between TFP and the prey cell is required for successful predation. However, it was not determined if TFP are required for the attachment to or invasion of a prey cell. These small (8 nm in width) polar fibers are incredibly strong and, as has been shown in *Pseudomonas aeruginosa* [4] and *Myxococcus xanthus* [5], have the ability to extend, adhere to a surface and retract. Successive cycles of extension, adherence and retraction is called twitching motility. The PilT protein, via ATP hydrolysis, powers the retraction of the pilus and can generate forces exceeding 100 pN per single fiber, making it one of the strongest biological motors [6]. Deletion of PilT in *P. aeruginosa* abolishes twitching motility and results in a hyperpiliated cell, unable to retract the pilus but still able to extend it. *B. bacteriovorus* contains two annotated *pilT* genes, *pilT1* and *pilT2* [7]. In a study of host-independent mutants of *B. bacteriovorus* 109J, Medina *et al.* [8] reported that transposon insertion into the *pilT* gene which encodes the motor which retracts the pilus (*pilT2* Bd3852) produced a mutant that had an impaired ability to prey on a preformed biofilm of *Escherichia coli* cells. Although predation in liquid cocultures was not included in this study, this result with biofilms suggested that *B. bacteriovorus* uses the retraction of TFP to pull itself into the periplasmic space of the prey.

Koval *et al.* [9] described an epibiotic predator, *Bdellovibrio exovorus*, which does not invade the periplasmic space of the prey but remains attached to the outer surface. Polar pili were demonstrated on the type strain *B. exovorus* JSS^T^ [3] and the genome of this strain contains a full set of genes encoding TFP including *pilT1* and *pilT2* [10]. *B. exovorus* does not require these two genes to invade the prey cell, and thus they must have some other function in the epibiotic life cycle. If *pilT1* and *pilT2* are essential for the epibiotic life cycle, mutants would need to be constructed in a prey-independent mutant. However, so far isolation of such mutants in *B. exovorus* JSS has not been successful [10]. Therefore, we undertook a study of the function of the *pilT1* and *pilT2* genes during the periplasmic life cycle of *B. bacteriovorus* 109J.

Markerless in-frame deletion mutants of *pilT1*, *pilT2* as well as a *pilT1pilT2* double deletion mutant were constructed in the prey-independent strain 109JA. All mutants produced bdelloplasts in cocultures with *E. coli* prey cells and thus the retraction of TFP is not required for successful invasion of a prey cell. These results, combined with previous studies on *pilA* mutants and the presence of TFP on the epibiotic predator *B. exovorus*, suggest that TFP are required for initial attachment to prey cells. This study also demonstrated that *pilT2* is required for efficient predation on a biofilm.

## Materials and Methods

### Bacterial strains, media and culture conditions

*E. coli* strains (Table 1) were grown routinely in LB medium at 30°C overnight. When required, cells were grown in LB containing kanamycin (50 µg/ml) or chloramphenicol (25 µg/ml). *B. bacteriovorus* 109J was maintained in coculture with *E. coli* ML35. Cocultures were prepared by mixing a 1∶3 ratio of predator to prey in HM buffer and incubated at 30°C overnight [11]. The facultative predator (prey-independent strain) *B. bacteriovorus* 109JA was grown in PY medium overnight at 30°C [3].

**Table 1:** Bacterial strains and plasmids used in this study.

Bacterial Strains	Source/Reference
*E. coli* ML35	Laboratory Strain
*E. coli* SY327λpir	Laboratory Strain
*E. coli* SM10 λpir	Laboratory Strain
*E. coli* CO1	Peter Cadieux, University of Western Ontario, London, Canada
*B. bacteriovorus* 109J	Laboratory Strain
*B. bacteriovorus* 109JA	John Tudor, St. Joseph's University, Philadelphia, USA
*B. bacteriovorus* 109JA::Δ*pilT1*	This study
*B. bacteriovorus* 109JA::Δ*pilT2*	This study
*B. bacteriovorus* 109JA::Δ*pilT1pilT2*	This study
*P. aeruginosa* PAK	Lori Burrows, McMaster University, Hamilton, Canada
*P. aeruginosa* PAK *+* pBADGr	This study
*P. aeruginosa* PAK *+* pBADGr::*pilT1*	This study
*P. aeruginosa* PAK *+* pBADGr::*pilT2*	This study
*P. aeruginosa* PAK::Δ*pilT*	Lori Burrows, McMaster University, Hamilton, Canada
*P. aeruginosa* PAK::Δ*pilT* + pBADGr	This study
*P. aeruginosa* PAK::Δ*pilT* + pBADGr::*pilT1*	This study
*P. aeruginosa* PAK::Δ*pilT* + pBADGr::*pilT2*	This study
**Plasmids**	
pSSK10	Silvia Piñeiro, University of Maryland, Baltimore, USA
pRCT1	This study
pRCT2	This study
pBADGr	Lori Burrows, McMaster University, Hamilton, Canada
pBADGr::*pilT1*	This study
pBADGr::*pilT2*	This study

### Construction of *B. bacteriovorus* 109JA markerless in-frame deletion mutants

Construction of the *pilT* mutants was performed in the facultative predator *B. bacteriovorus* 109JA because these gene deletions have the potential to be lethal. The *pilT1* gene (locus tag Bd1510) and the *pilT2* gene (locus tag Bd3852) were knocked out by allelic exchange using an in-frame deletion cloned on the suicide plasmid pSSK10. Deletion constructs were prepared as described in Steyert and Piñeiro [12] using primers listed in Table 2 with modification of the restriction sites used. A *Spe*I site was used to ligate the upstream and downstream fragments together. *Nde*I and *Xho*I were used for the upstream and downstream fragments respectively, to directionally ligate into pSSK10.

**Table 2:** PCR oligonucleotide primers used for amplification of *pilT1* and *pilT2*.

Primer	Sequence (5′–3′)	Target Gene
PilT1-F1	ATAGTAACATATGACGTGAACATCTCCACCG	*pilT1*
PilT1-R1	AATAGCATGCATTCCGTCATCAGCGCTG	*pilT1*
PilT1-F2	ACATGCATGCAACATCTGATTCGTCGTCG	*pilT1*
PilT1-R2	ATACTCGAGACCGGAACCAGCGAAGAAT	*pilT1*
		
PilT2F1	CACACATATGGTCTTCGATACGACGGGAAA	*pilT2*
PilT2R1	AATAGCATGCGTTCAATGGAACCATGTTTC	*pilT2*
PilT2F2	AATCGCATGCACCAAAGTGGGCTAA	*pilT2*
PilT2R2	ATACTCGAGAAGTTCCGCGCAGGTCTT	*pilT2*

*restriction sites are underlined.

The correct construction of the plasmids pRCT1 and pRCT2 was confirmed by sequencing. *E. coli* SM10*λpir* harboring either pRCT1 or pRCT2 was used as a donor for conjugation into the recipient strain 109JA using the method of Cotter and Thomashow [13]. Dilutions were plated onto 1% PY agar containing streptomycin to select for *B. bacteriovorus* 109JA and chloramphenicol to select for the presence of plasmid. The presence of merodiploid strain 109JA in the cultures was confirmed by PCR using the same outer flanking primers that had been used for cloning. An aliquot of a merodiploid culture was added to fresh PY with no antibiotic and grown for 24 h at 30°C. This was repeated 3 times to allow for excision of the suicide plasmid by a second homologous recombination event. Counterselection was performed using PY containing 5% sucrose to select for excisants. Dilutions were plated and colony PCR was performed to identify individual mutants.

### Liquid predation assay

Liquid coculture predation assays were performed in triplicate using a 100-well BioScreen plate incubated for 48 h at 30°C. Samples were prepared by growing *B. bacteriovorus* 109JA and mutants axenically in PY medium (5% v/v inoculum) with shaking at 30°C. An aliquot of cells from each culture was washed and resuspended in 1 ml of HM buffer. The optical density at 600 nm (OD_600_) was used to normalize the number of cells used for individual experiments.

An aliquot of each predator sample was normalized to have an OD_600_ of 0.2. One millilitre was centrifuged at 2500 *g* for 5 min, resuspended in HM buffer and added to a 20 ml coculture. A 16 h culture of *E. coli* ML35 was concentrated in HM buffer and added to obtain a coculture with an OD_600_ reading of 1.0. Two hundred microlitres were added to each well and readings were taken every 30 min for a total of 48 h.

### Biofilm predation assay

Using a protocol modified from Kadouri and O'Toole [14], biofilms were formed in round-bottom microtiter dishes (BD Falcon). Microtiter wells were inoculated (200 µl per well) from 18 h *E. coli* LB-grown cultures diluted 1∶100 in LB. Cells were grown for 48 h at 30°C to allow the biofilm to form. Quantification of biofilm bacteria was performed as follows. The wells were washed 3 times with HM buffer in order to remove any planktonic cells and 200 µl of crystal violet was then added for 15 min. Wells were washed with ddH_2_0 4 times and allowed to dry. Crystal violet was solubilized by adding 200 µl of 30% acetic acid for 20 min. An aliquot (125 µl) of each well was transferred to a flat-bottom microtiter dish (BD Falcon) and analyzed using a microplate reader at 600 nm. Preliminary studies confirmed the use of *E. coli* CO1 as the best candidate for biofilm studies (Fig. S2).

To assess predation of *B. bacteriovorus* on *E. coli* biofilms, the preformed biofilms were grown as described above and washed three times with HM buffer to remove planktonic cells. *B. bacteriovorus* cultures grown in PY overnight were washed and diluted 1∶100 in HM buffer and 200 µl added to each well. As a control, 200 µl of a filtered sterilized lysate was prepared by passing the *B. bacteriovorus*-containing lysate through a 0.22 µm pore size filter. After filtering, no predator could be detected as judged by setting up a coculture as described earlier. The microtiter dish was incubated at 30°C for 24 h. Quantification of biofilm was performed as described above. Data presented were an average of 12-wells per replicate repeated three times. Statistical significance was measured using a 1-way ANOVA with a Bonferonni corrected post-hoc Students T-test, *p<0.005.

### Immunofluorescence Microscopy

Immunofluorescence microscopy was used to count the number of cells which had TFP. Cells of strain 109JA and the *pilT* mutants were grown as described earlier. An aliquot (200 µl) was pre-fixed with 0.25% paraformaldehyde in Dulbecco's buffered saline (DPBS, 2.7 mM KCl, 1.5 mM KH_2_PO_4_, 136.9 mM NaCl and 8.9 mM Na_2_HPO_4_•7H_2_O) and centrifuged at 5000 *g* for 5 min. Samples were resuspended in full strength fixative (2.5% paraformaldehyde in DPBS) and incubated at 37°C for 10 min before being washed twice in 2.5% bovine serum albumin in DPBS (BSA-DPBS). Samples were resuspended in 1∶100 anti-pilA antibody [3] in BSA-DPBS, incubated for 1 h at 37°C then washed twice in BSA-DPBS. Antibody detection was performed by resuspending samples in 1∶100 sheep anti-rabbit IgG conjugated to Cy3 in BSA-DPBS, incubated for 1 h at 37°C and washed three times in BSA-DPBS. Samples were visualized by placing 10 µl onto CELLSTAR microscope slides and viewed using an Axioskop II epifluorescence microscope (Zeiss) equipped with a QImaging Retiga 1300 cooled monochrome 12-bit camera and an HBO100/2 mercury lamp for epifluorescence illumination. Imaging was performed using Northern Eclipse software version 6.0 (Empix Imaging Inc.).

### Electron Microscopy

To visualize TFP, cells were negatively stained and viewed by transmission electron microscopy (TEM). Cells were negatively stained on a 400-mesh Formvar-carbon-coated copper grid that was inverted over a drop of cells for 1 min. The grid was then washed on 2 drops of water and the cells were stained with 1% uranyl acetate containing bacitracin (50 µg/mL) as a wetting agent. Negative stains were visualized using a Philips EM 410 transmission electron microscope operating at 60 kV.

To visualize biofilms, scanning electron microscopy (SEM) was used. *E. coli* CO1 biofilms were developed on a 12-by-22-mm PVC plastic coverslip (Fisher Scientific, Pittsburgh, PA). The coverslips were placed in a 6-well polystyrene cell culture plate (Corning, Inc., Corning, NY) and inoculated with a 1∶100 dilution of a culture of *E. coli* CO1 in LB. Plates were incubated at 30°C for 48 h with shaking. Preformed biofilms were rinsed three times in a buffer consisting of equal parts 0.07 M sodium phosphate dibasic and 0.07 M potassium phosphate monobasic (SEM buffer, pH 6.8) to remove any planktonic cells. *B. bacteriovorus* 109JA and the *pilT* mutants were prepared as described above for previous biofilm experiments and added to an appropriate well. The biofilms were incubated at 30°C with shaking (120 rpm) for 24 h before being rinsed three times in SEM buffer. Residual biofilms were fixed for 30 min in SEM buffer containing 2% glutaraldehyde, followed by another three washes in SEM buffer and a 30 min secondary osmium fixation (2% osmium tetroxide). The samples were washed in sterile water and dehydrated step-wise in an ethanol series (70%, 80%, 90%, 3×100%). Each step was left to sit for 10 min. The samples were critically point dried at the Biotron Integrated Microscopy Facility (University of Western Ontario, London, ON, Canada). The samples were platinum coated and visualized on a Hitachi S-4500 field emission scanning electron microscope operating in high vacuum using Quartz XOne version 9.50 imaging software at the Surface Science Facility (University of Western Ontario, London, ON, Canada). Scanning electron microscopy was repeated on two separate occasions.

## Results

### Comparative analysis of active domains in *pilT* genes of *Bdellovibrio bacteriovorus*

The number and chromosomal arrangement of TFP genes is the same in *B. bacteriovorus* strains HD100 and 109J [3]. As described by Mahmoud and Koval [3], *pilT1* (Bd1510) is located within an operon containing *pilB* upstream and *pilC*, *pilS* and *pilR* directly downstream. It does not contain a Walker A box necessary for binding ATP. In contrast, *pilT2* (Bd3852) contains the Walker A (GPTGSGKS) box and the two turn amphipathic α-helical AIRNLIRE sequence important for its function [15]. The *pilT2* gene is not found within an operon and is not close to any other genes involved in TFP assembly, regulation or function.

In this study, a closer analysis of *pilT1* revealed that, although it does not contain a recognized nucleotide-binding site, it does contain a Walker B box. However this would be insufficient for binding ATP. It does not contain any other domains recognized in the PilT proteins of *P. aeruginosa* or *Neisseria gonorrhoeae* that are important for function. Because of this, PilT1 may not be involved in the retraction process but may function in regulation or stability of the pilus, similar to PilU of *P. aeruginosa* [16].

Analysis of PilT2 revealed that it contains both the Walker A and the Walker B domains. It also contains many of the active domains found in PilT of *P. aeruginosa* with minor differences in amino acid composition. There was a minor variation in the AIRNLIRE sequence, with two amino acid differences (AISNLVRE). It is believed this sequence may be involved in the regulation of PilT function and does not contain catalytic activity. Although both isoleucine residues are essential for PilT function [15], an isoleucine to valine substitution is generally regarded as a homologous substitution and should not inhibit proper function of PilT2. The Asp Box (EDPIE) is identical to that found in *P. aeruginosa* while the His Box (HLVFGTVH) has a single leucine to isoleucine substitution. Structural studies of the PilT gene in *Aquifex aeolicus* and *Vibrio cholerae* identified the ASP and His boxes as forming the catalytic pocket [17]. Therefore, PilT2 contains the domains which bind ATP, form the catalytic pocket and is capable of regulating its own function. A mutant lacking this gene should not be able to retract its pilus and thus would help to determine if *B. bacteriovorus* uses PilT2 to generate the force necessary for the invasion process.

### Growth and Predation Assay of *pilT* mutants

The facultative predator *B. bacteriovorus* 109JA was used to create *pilT* mutants because these genes were hypothesized to be essential for predation. The ability of the *pilT* mutant to grow axenically as well as a predator could then be tested.

**Figure 1:**
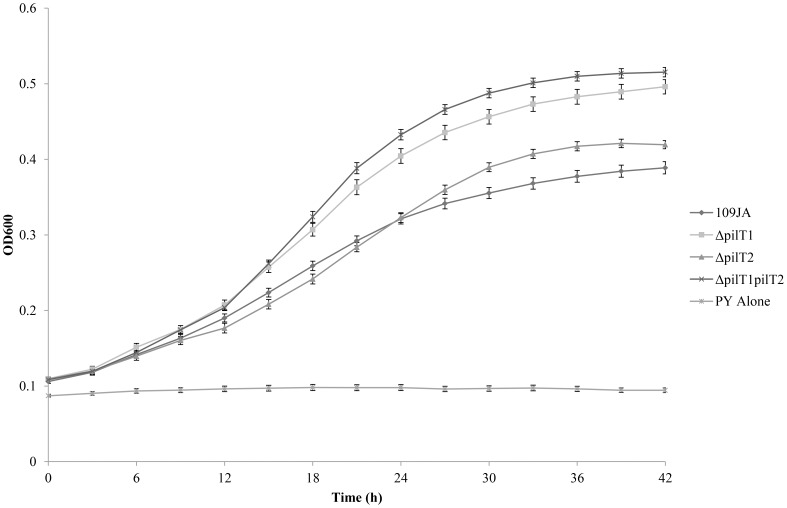
Axenic growth curve of *B. bacteriovorus* 109JA and *pilT* mutants. Growth in PY medium over a 42 h period. Results shown are an average of 20 replicates repeated in triplicate. Error bars represent standard error.

In axenic growth mode, wild type strain 109JA and the *pilT2* mutant grew in PY medium at the same rate reaching a final OD_600_ of 0.4 after 36 h (Fig. 1). The *pilT1* mutant and the *pilT1pilT2* double mutant also grew at similar rates, reaching a final OD_600_ of 0.5 after 36 h. After 30 h, growth of all strains began to plateau and there was no further increase in optical density. Examination by light microscopy did not reveal any variation in cellular morphology which would account for the increased optical density of cultures of the two mutants, such as elongated, undivided or corkscrew shaped cells which are occasionally observed during axenic growth of these predators. Nor is the lower optical density of wild type and the *pilT2* mutant cultures due to aggregation of cells. In all three mutants and the wild type, cells became elongated and occasionally formed a corkscrew shape if left in medium for longer than 48 h.

**Figure 2:**
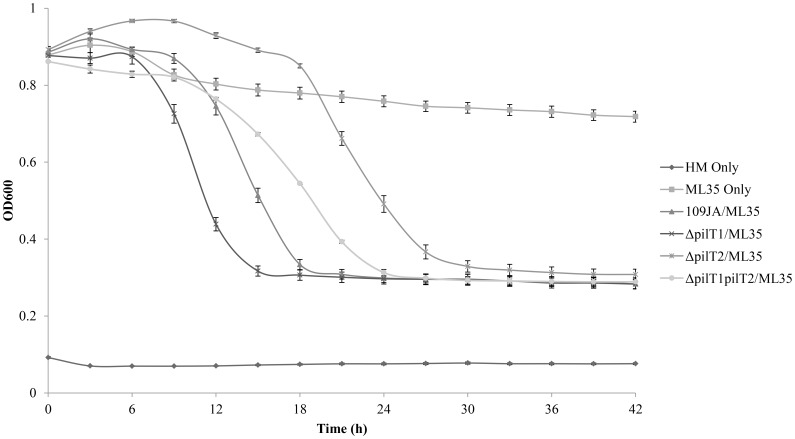
Effect of mutations in *pilT* genes on predation by *B. bacteriovorus* on *E. coli.* The decrease in prey cell optical density was used to assess predator growth. Results shown are an average of 20 replicates repeated in triplicate. Controls included: HM Buffer alone, *E. coli* ML35 prey cells alone and the wild type strain 109JA. Error bars represent standard error.

In predatory mode, *B. bacteriovorus* 109JA preyed efficiently and produced bdelloplasts on *E. coli* ML35. Maximum growth was reached after 18 h as measured by a decrease in prey cell turbidity (Fig. 2). The *pilT1* mutant reached maximum growth after only 15 h. The *pilT2* mutant grew more slowly, reaching the maximal decrease in optical density after 30 h. The *pilT1pilT2* double mutant preyed within 24 h which was slower than the *pilT1* mutant but quicker than the *pilT2* mutant, restoring it to a near wild type predation pattern. All *pilT* mutants produced bdelloplasts of *E. coli* (as judged by phase contrast light microscopy; Fig. S1). Although minor variations in predation kinetics were observed (Fig. 2), overall neither PilT1 nor PilT2 were required for invasion of prey cells.

### Presence of Type IV Pili on *pilT* mutants

Western blot analysis of whole cell lysates of axenically grown strain 109JA and the *pilT* mutants showed similar levels of the PilA protein present (Fig. S3). To examine the piliation of individual cells, electron microscopy and immunofluorescence light microscopy were used.

Electron microscopy evaluation of strain 109JA showed that 22.3% (n = 336) of cells expressed TFP on their surface (Table 3). This was significantly (p<0.005) different than the *pilT1* mutant, of which only 8.2% (p<0.005, n = 346) of cells were observed to have TFP on their surface. Only 16.5% (n = 250) of the *pilT2* mutant cells were piliated. Surprisingly, the *pilT1pilT2* double mutant had near wild type levels with 21.2% (n = 330) piliation.

**Table 3:** Presence of type IV pili on the surface of cells as assessed by electron microscopy and immunofluorescence microscopy.

Strain	% Number of cells with TFP	
	Electron Microscopy	Immunofluorescence
109JA	22.3	27.0
Δ*pilT1*	8.2*	33.4*
Δ*pilT2*	16.5*	29.2*
Δ*pilT1pilT2*	21.2	27.7

Statistical significance determined using the Chi-Square test, *p<0.005.

None of the mutants were hyperpiliated as is observed with *P. aeruginosa* (Fig. S3B). In our study, the TFP were always located on the non-flagellated pole. In the rare instance that the cell did not have a flagellum, TFP were never observed on both poles. The number of TFP per cell (one) did not appear to change, however, this could only be assessed by electron microscopy. On occasion a cell was observed to have more than one pilus (Fig. S4).

Due to the short length of TFP on *B. bacteriovorus* (Fig. S4), staining artefacts and the laborious task of finding cells that were stained optimally for visualizing and thus counting TFP, electron microscopy was not an ideal method for determining the level of piliation. Therefore immunofluorescence microscopy with antibodies to the PilA protein was used for a more accurate measurement. In all cases (wild type and mutants) the number of cells with TFP increased. Both the wild type and the *pilT1pilT2* mutant had about 27% of cells with TFP (n = 4308, n = 3103). Surprisingly, the *pilT1* mutant had 33.4% (n = 5252) piliation, a significant (p<0.005) increase from 8.2% measured by electron microscopy. The *pilT2* mutant had 29.2% of cells (n = 3073) with TFP, a small but significant increase above wild type levels (p<0.005).

### Biofilm Predation Assay

**Figure 3:**
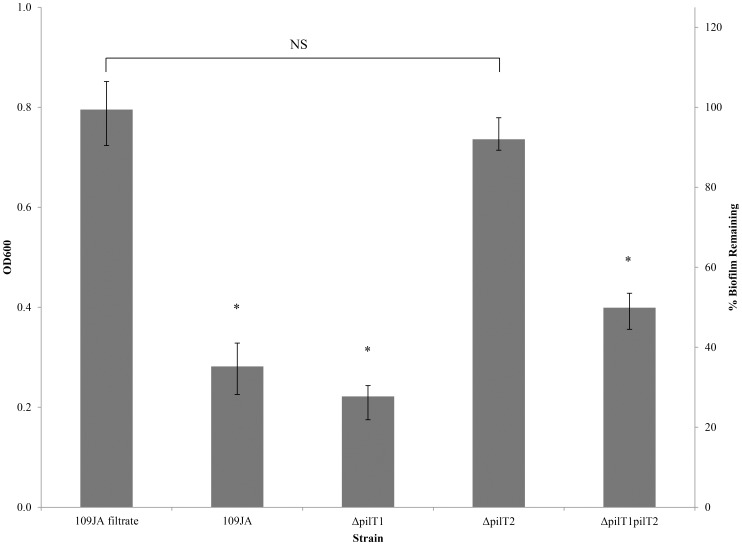
Biofilm predation assay. Biofilms of *E. coli* CO1 were pre-formed for 48 h in 96-well microtiter plates. Predator cultures containing either *B. bacteriovorus* 109JA or a *pilT* mutant were added and the plates incubated for a further 24 h. Residual biofilm cells were stained with crystal violet and the optical density at 600 nm (OD600) determined (*p<0.005). To exclude secreted factors contributing to the decrease in remaining biofilm, a 0.45 µm filtrate of a *B. bacteriovorus* 109JA culture used as a control. The percent biofilm remaining relative to the 109JA filtrate is shown on the secondary axis. Data presented were an average of 12-wells per replicate repeated in triplicate. Statistical significance was measured using a 1-way ANOVA with a Bonferonni corrected post-hoc Students T-test, *p<0.005.

During this study, it was noted that the mutant with the markerless in-frame deletion of *pilT2* did not form plaques on lawns of *E. coli*, however the *pilT1* and *pilT1pilT2* mutants did (Data not shown). This observation confirmed the results of Medina *et al.* [8], who reported that a *pilT2* transposon mutant could not form lytic halos on lawns of *E. coli*. Therefore a robust biofilm model was used to assess predation of these predators. *E. coli* CO1 was used as the prey cell as it is a known biofilm-producing strain isolated from the stool of a healthy woman. This strain showed an increased ability to attach to uroepithelial cells of the elderly and agglutinate P-type erythrocytes, indicating it has potential to cause renal infections [18]. This strain was chosen over other known biofilm producers such as *P. aeruginosa* and *E. coli* strains GR12 and C1212 as it formed the best biofilm under the experimental conditions tested to allow for *Bdellovibrio* growth (Fig. S2). Wild type *B. bacteriovorus* 109JA reduced the mass of the biofilm by 65% while the *pilT2* mutant was unable to prey on the biofilm (Fig. 3). The *pilT1* mutant reduced the biofilm mass by 72%, slightly more than wild type which coincides with results observed in liquid cocultures where the *pilT1* mutant was slightly more aggressive in predation. Surprisingly, the *pilT1pilT2* double mutant was able to prey on the biofilm, but only able to reduce the mass by 49%. This indicates that although *pilT2* is required for predation on the biofilm, the *pilT1* deletion is able to partially restore function.

### Scanning Electron Microscopy

**Figure 4:**
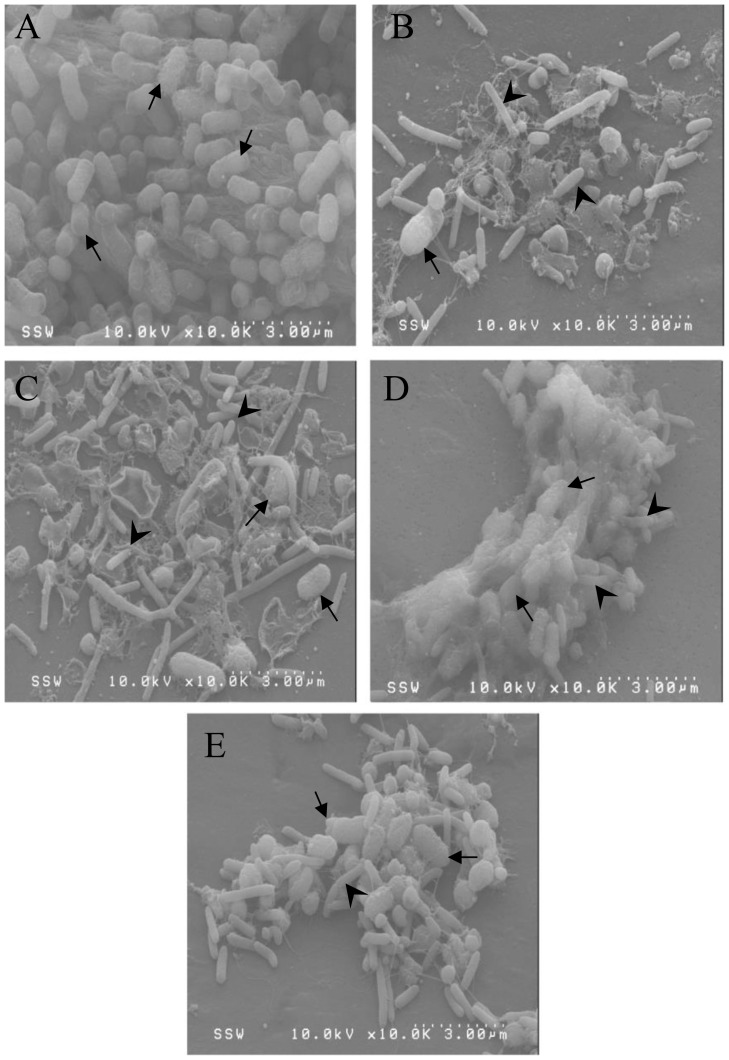
Scanning electron microscopy of predation on pre-formed biofilms of *E. coli*. (**A**) Biofilms of *E. coli* CO1 (arrows) were formed for 48 h on polyvinyl chloride plastic coverslips. Predator cultures (arrowheads) of (B) *B. bacteriovorus* 109JA, (C) Δ*pilT1*, (D) Δ*pilT2* (E) Δ*pilT1pilT2* were added for a further 24 h.

Biofilm formation is a complex process which involves the secretion of many factors to create the extracellular polymeric substance (EPS). The EPS secreted by *E. coli* CO1 was seen as a dense, fibrous matrix which protects the cells beneath (Fig. 4A). Cells on the surface are not as protected as those buried beneath the EPS. *B. bacteriovorus* 109JA was able to penetrate the EPS layer and disrupt the biofilm. An overall loss in surface biofilm combined with the observation of bdelloplasts and many attack phase *B. bacteriovorus* (Fig. 4B) suggested that the EPS was not a barrier to predation. The *pilT1* mutant was able to disrupt the biofilm (Fig. 4C), but less predation was observed with the *pilT2* (Fig. 4D) and the double mutant (Fig. 4E). The biofilm exposed to the *pilT2* mutant had more EPS surrounding the prey cells than that seen when the biofilm was exposed to the *pilT1pilT2* mutant. These results suggest that the *pilT1pilT2* mutant can kill prey cells in a biofilm but the *pilT2* mutant has not been able to penetrate and disrupt the EPS.

## Discussion

The mechanism which facilitates invasion of the prey cell periplasmic space by *B. bacteriovorus* is beginning to be understood. Publication of the genome sequence of *B. bacteriovorus* in 2004 allowed us to speculate which genes may be important for this process. Evans *et al*. [2] and Mahmoud and Koval [3] demonstrated that TFP are essential for predation to occur. Deletion of the gene for the major pilin protein, PilA, resulted in non-predacious *B. bacteriovorus*. Mahmoud and Koval [3] raised antibodies against the PilA protein and showed that the presence of anti-PilA in cocultures was enough to delay and inhibit predation. No attachment between *B. bacteriovorus* and the prey cell was observed for 16 h, suggesting that TFP are important for attachment to prey cells. However, because TFP in other bacteria are able to extend and retract, it was necessary to ask the question: Is retraction of TFP the driving force behind prey cell entry? Amino acid sequence analysis of PilT2 showed high sequence identity to other retraction ATPases, and thus it is suggested that this gene encodes an ATPase that could retract TFP in *B. bacteriovorus.* This study has demonstrated that PilT1 and PilT2 are not required for predation in liquid cocultures and, more specifically, they are not required for invasion of prey cells.

Bioinformatic analysis of the active domains present in PilT1 of *B. bacteriovorus* revealed that although it was annotated as a homologue of a PilT protein, it does not appear to function in this regard. It may be more similar to PilU in *P. aeruginosa*, which is thought to increase pilus strength or stability [16]. This may account for the decrease in number of TFP assessed by negative staining of the *pilT1* mutant. This staining method may possibly shear the pilus during the preparation process, giving the appearance of decreased surface associated TFP.

Bioinformatic analysis of PilT2 demonstrated high conservation within the active domains among other PilT proteins known to retract TFP, such as *P. aeruginosa* and *N. gonorrhoeae*. Twitching motility could not be demonstrated in *B. bacteriovorus* 109JA or the *pilT* mutants by use of a conventional sub-surface twitching motility assay designed for studies with *P. aeruginosa* [19]. It is possible that the prey-independent strain used in our study (strain 109JA) is unable to use twitching motility. The obligate predatory life cycle of wild type strains does not allow for twitching motility to be directly measured using this method. Expression of *B. bacteriovorus* PilT1 or PilT2 in a *P. aeruginosa* PilT deletion mutant was unable to restore the twitching phenotype (Fig. S5). Lambert *et al.* [20] described *Bdellovibrio* gliding motility, a type of surface motility not flagellar or pilus mediated. In our study, the *pilT* mutants were still able to use gliding motility, confirming this is independent of TFP and PilT1 or PilT2 (Data not shown).

It is an unresolved question as to why so few cells of *B. bacteriovorus* express TFP on their surface in *in vitro* cocultures, yet are essential for survival. It is not known in a batch culture what percentage of *B. bacteriovorus* cells are capable of predation. It is possible that only a minority of the population are able to prey and therefore only this minority would express TFP. Both the *pilT1* and *pilT2* mutants showed a small increase in the number of cells with TFP as assessed by immunofluorescence microscopy. This result suggests that these genes may be involved in TFP function but does not elucidate what role each gene is playing, whether it be in regulation or direct involvement.

The ability of *B. bacteriovorus* to disrupt and kill cells within a biofilm has been studied previously [14], [21], [22]. We have shown that *pilT2* is not required for efficient predation in liquid coculture but is required for predation on a biofilm, confirming the results of Medina *et al.* [8]. SEM analysis revealed that an EPS layer is produced by *E. coli* cells during biofilm formation. This layer adds a level of protection from the outer environment but not to *B. bacteriovorus* predation, as predators were capable of penetrating and disrupting the biofilm. However, the *pilT2* and *pilT1pilT2* mutants were not able to do this as efficiently. It appears predation is able to occur on the cells at the surface of the biofilm. Eventually other cells would be preyed upon, but this would require a higher concentration of *Bdellovibrio* or more time. Taken together, the delay in predation on biofilms by *pilT2* and *pilT1pilT2* mutants is possibly due to the inability to retract their TFP via inactivation of the *pilT2* gene. The *pilT1* mutant still disrupted the biofilm and also partially restored the ability of the *pilT1pilT2* mutant to prey on the biofilm. It is known that *B. bacteriovorus* secretes hydrolytic enzymes such as proteases into the culture supernatant and that some of these enzymes have the ability to disrupt biofilms of Gram-positive pathogens such as *Staphylococcus aureus* [23]. The regulation of production of such enzymes is unknown and possibly could be signalled through the TFP system. Removal of the EPS layer in a biofilm by proteases could expose the underlying prey cells to predation. If PilT1 is a negative regulator of protease synthesis, then deletion of *pilT1* would result in an upregulation of enzyme production. This may explain why, although not statistically significant, deletion of *pilT1* was able to prey slightly better in liquid cocultures (Fig. 2) and on biofilms (Fig. 3). Also, it partially restored predation of the *pilT1pilT2* mutant closer to wild type in both predation assays. An increase in secreted hydrolytic enzymes may prime *B. bacteriovorus* for predation and allow easier entry into the prey cell.

The genome sequence of the epibiotic predator, *B. exovorus* JSS, contains a fully annotated set of TFP genes, including both *pilT1* and *pilT2* [10]. In this species invasion does not occur, an observation which supports the view of this study that retraction of TFP is not the driving force behind the invasion process. A PilT2 deletion in *B. exovorus* could not be isolated as it would not form plaques on an agar surface. To avoid this problem prey-independent strains are generally used. However, our lab and that of Jurkevitch [10] have been unable to produce a prey independent derivative of *B. exovorus* JSS.

The ability of most BALOs to penetrate the outer membrane of a Gram-negative cell without membrane fusion, in direct opposition of the internal turgor pressure of the prey cell, still remains an enigma. Other mechanisms must be at play to allow this event to occur. Pasternak *et al.* [10] hypothesized that the epibiotic predator *B. exovorus* evolved from a periplasmic predator by gene loss, including the loss of the genes that enable prey invasion. The identification of such genes would be step forward in understanding the invasion process.

## Supporting Information

Figure S1 **Phase contrast micrograph of predation of** ***pilT2*** **mutant (white arrow) on** ***E. coli*** **ML35.** Bdelloplasts (black arrow) and uninfected *E. coli* prey cells (black arrowhead).
(TIF)

Figure S2 **Prey cell biofilm formation.** Variation in biofilm formation of *E. coli* strains was assessed by staining residual cells with crystal violet. *E. coli* CO1 produced the best biofilm under the experimental conditions used.
(TIF)

Figure S3 **PilA western blot analysis.** The level of PilA (19.6 kDa) in whole cell lysates of *B. bacteriovorus* 109JA and the *pilT* mutants grown prey-dependently on *E. coli* ML35 was assessed.
(TIF)

Figure S4 **Electron micrographs of type IV pili (arrows) of (A)** ***B. bacteriovorus*** **109J and (B)** ***P. aeruginosa*** **PAK::** ***pilT*** **.** Cells were negatively stained with uranyl acetate. Note the hyperpiliated phenotype of the *P. aeruginosa* mutant.
(TIF)

Figure S5 **Subsurface twitching motility assay.** *P. aeruginosa* PAK and a PilT mutant expressing PilT1 or PilT2 from *B. bacteriovorus* were used to assess twitching motility. PilT proteins were expressed on an arabinose inducible plasmid (pBADGr) and the *P. aeruginosa* cultures plated on LB agar containing 0.1% arabinose. As a control, 0.2% glucose was added to repress expression. The zone of motility was visualized using crystal violet and the diameter of each zone measured.
(TIF)
