# Supplementary figures and images for: Role of Type IV Pili in Predation by Bdellovibrio bacteriovorus
Source: PLoS One. 2014 Nov 19;9(11):e113404. doi: 10.1371/journal.pone.0113404 (PMC4237445; doi:10.1371/journal.pone.0113404)

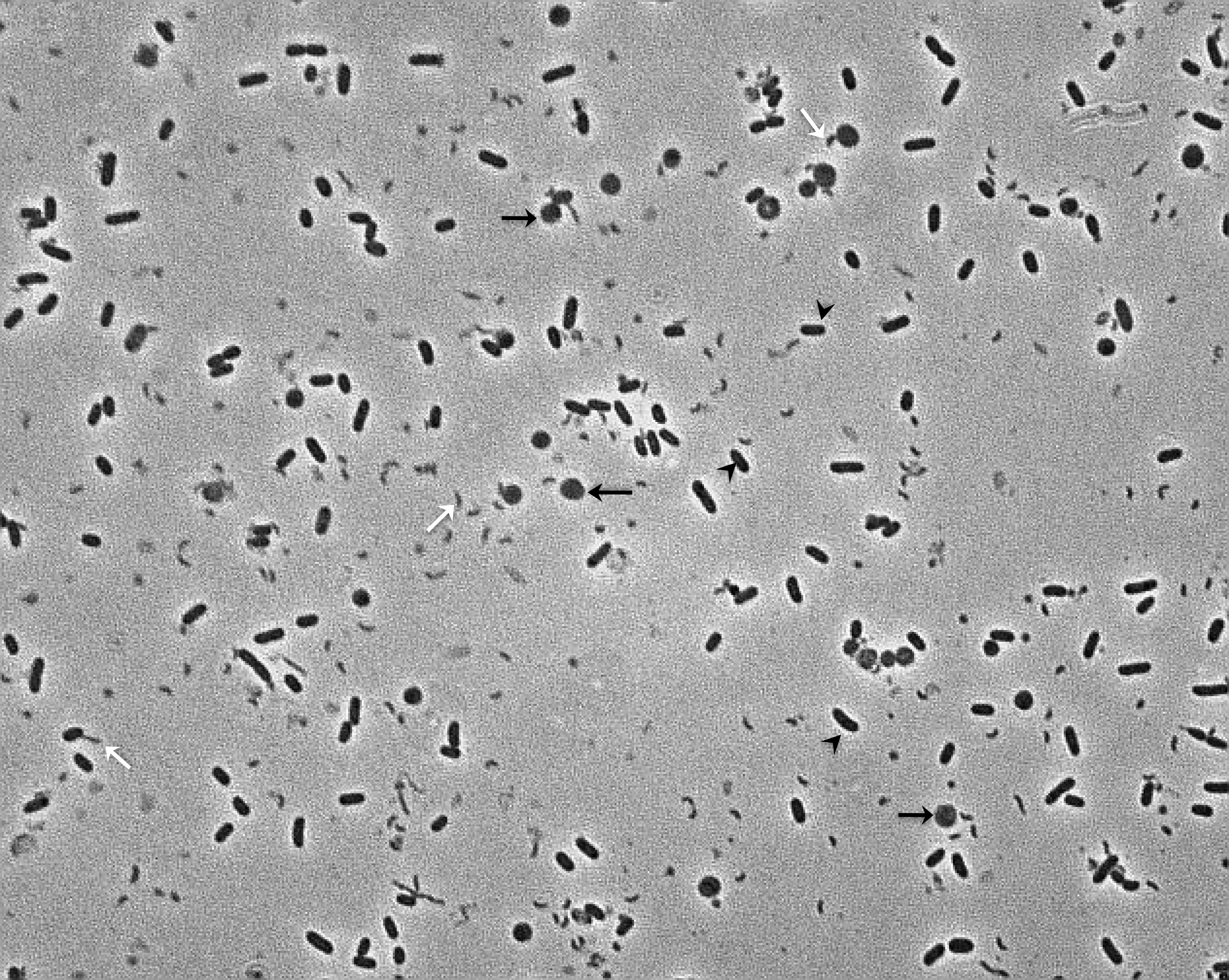

Supplement: Figure S1 — Phase contrast micrograph of predation of pilT2 mutant (white arrow) on E. coli ML35. Bdelloplasts (black arrow) and uninfected E. coli prey cells (black arrowhead). (TIF) [file pone.0113404.s001.tif]

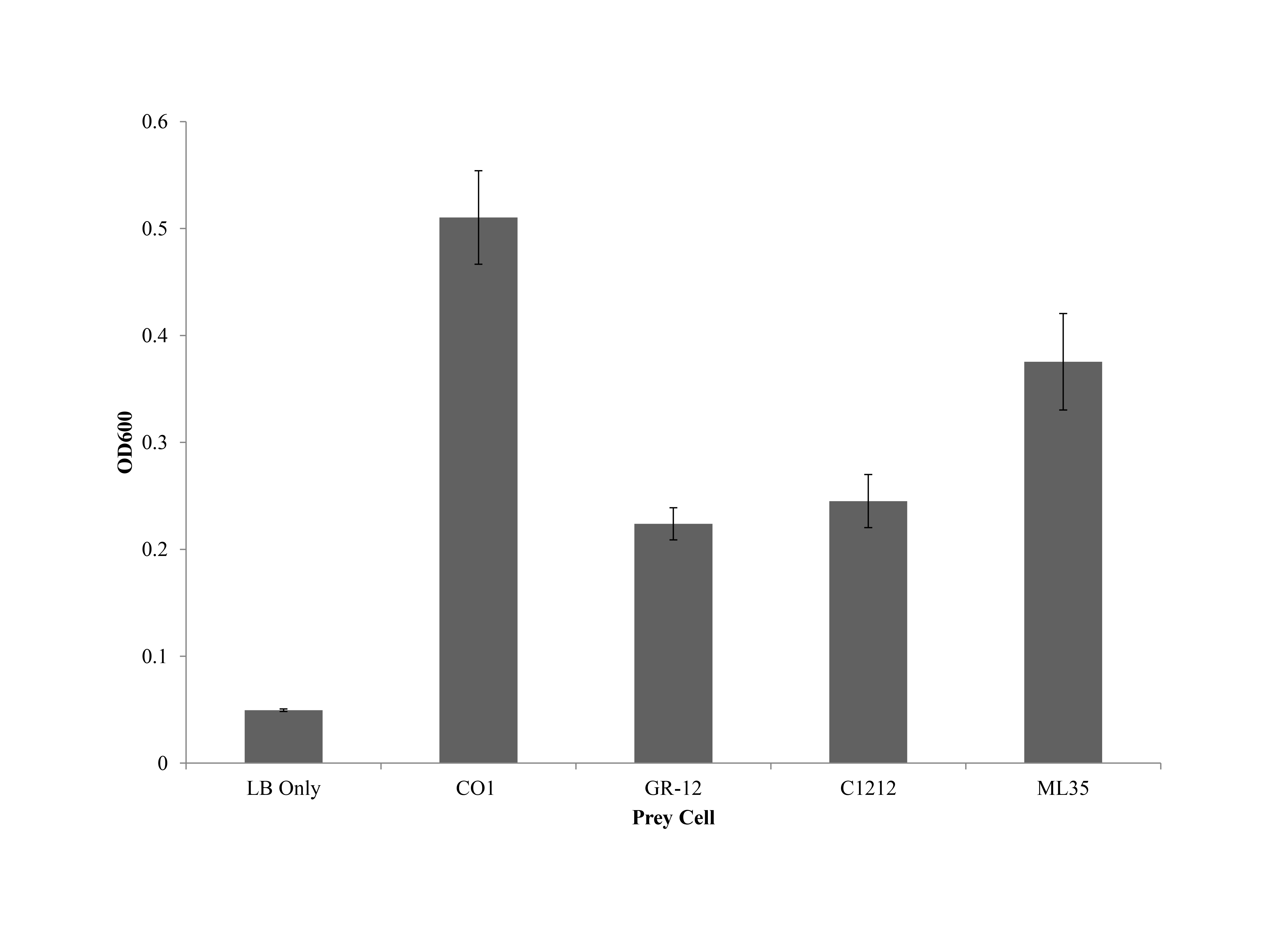

Supplement: Figure S2 — Prey cell biofilm formation. Variation in biofilm formation of E. coli strains was assessed by staining residual cells with crystal violet. E. coli CO1 produced the best biofilm under the experimental conditions used. (TIF) [file pone.0113404.s002.tif]

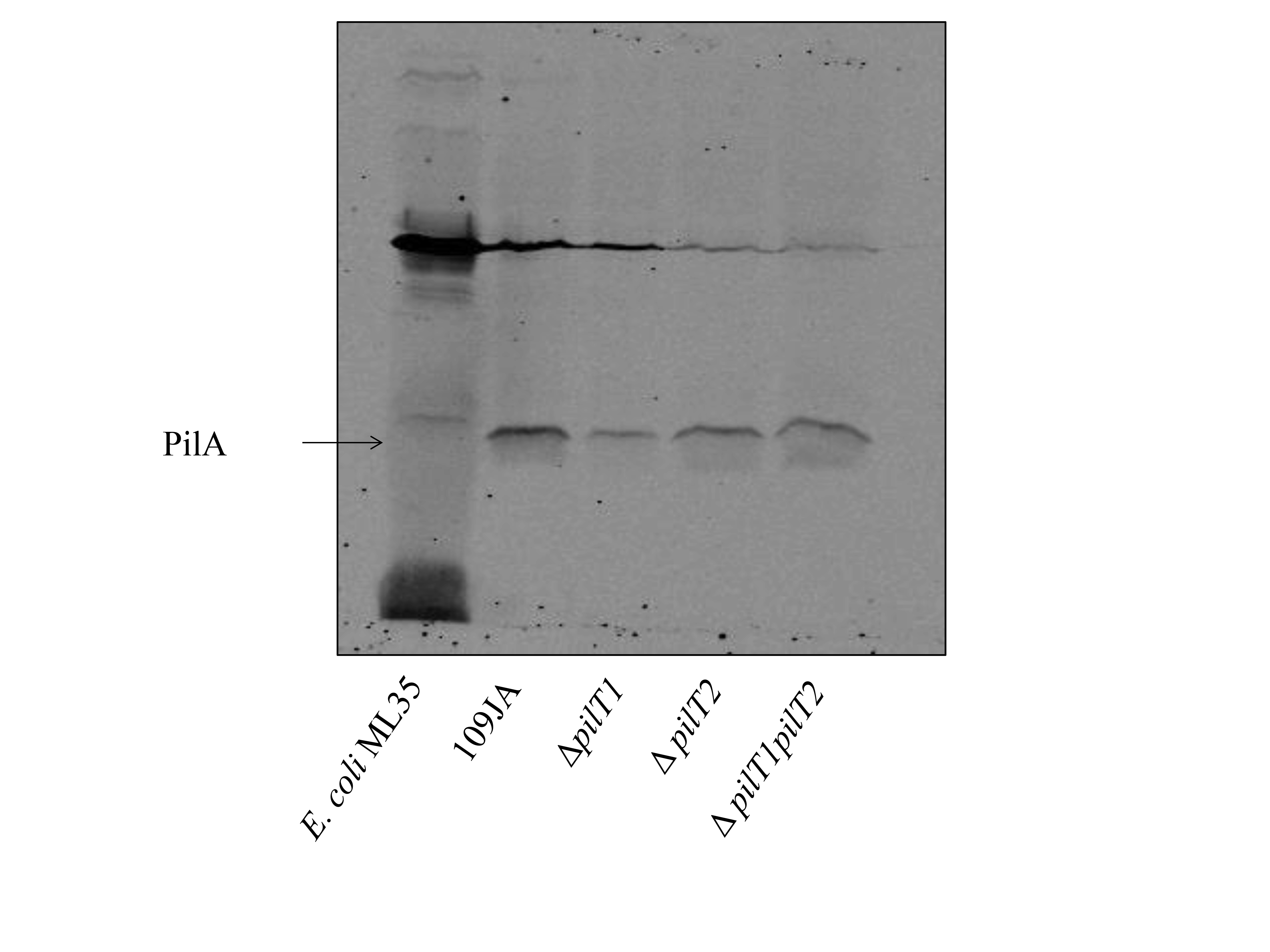

Supplement: Figure S3 — PilA western blot analysis. The level of PilA (19.6 kDa) in whole cell lysates of B. bacteriovorus 109JA and the pilT mutants grown prey-dependently on E. coli ML35 was assessed. (TIF) [file pone.0113404.s003.tif]

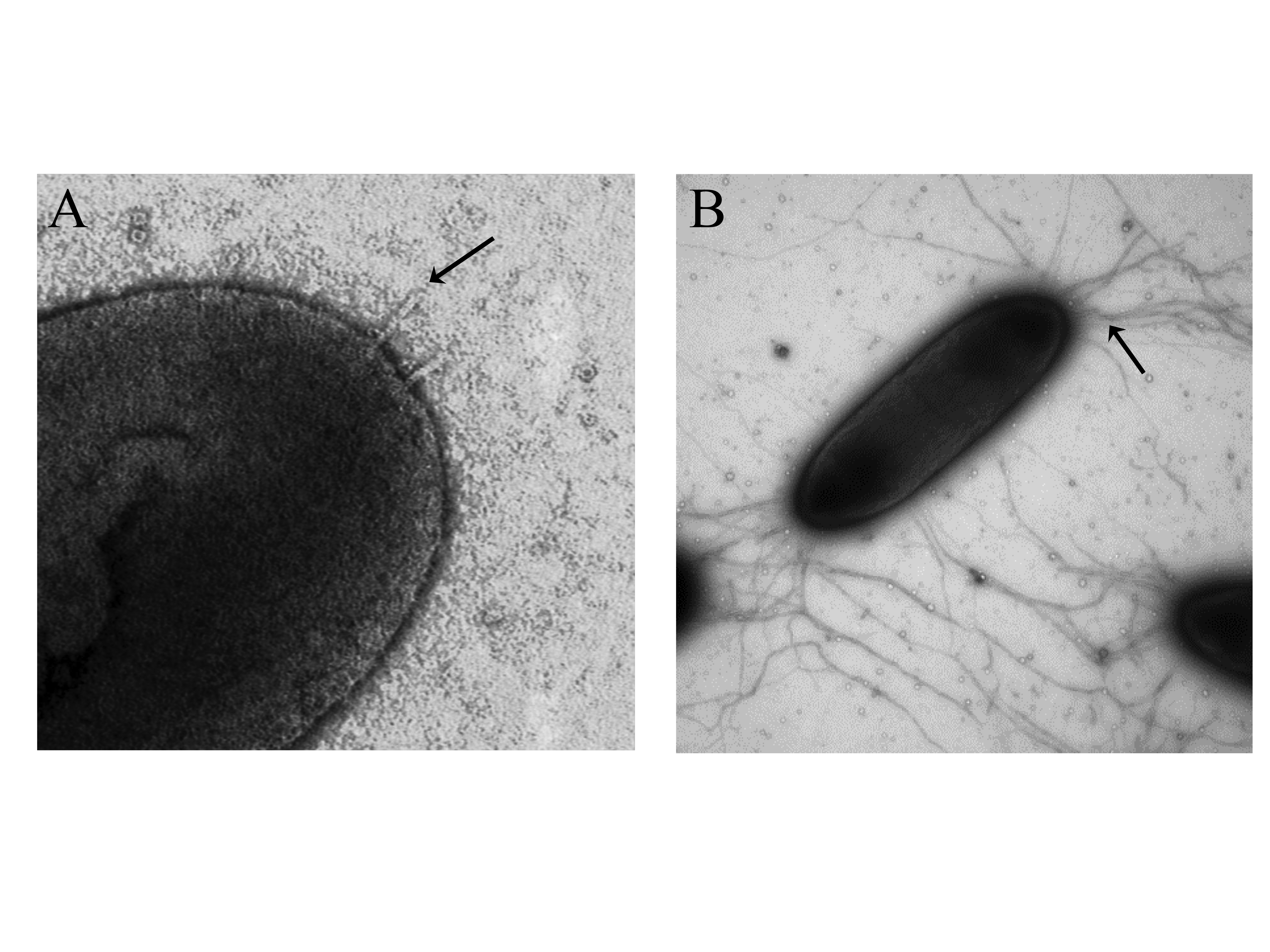

Supplement: Figure S4 — Electron micrographs of type IV pili (arrows) of (A) B. bacteriovorus 109J and (B) P. aeruginosa PAK:: pilT . Cells were negatively stained with uranyl acetate. Note the hyperpiliated phenotype of the P. aeruginosa mutant. (TIF) [file pone.0113404.s004.tif]

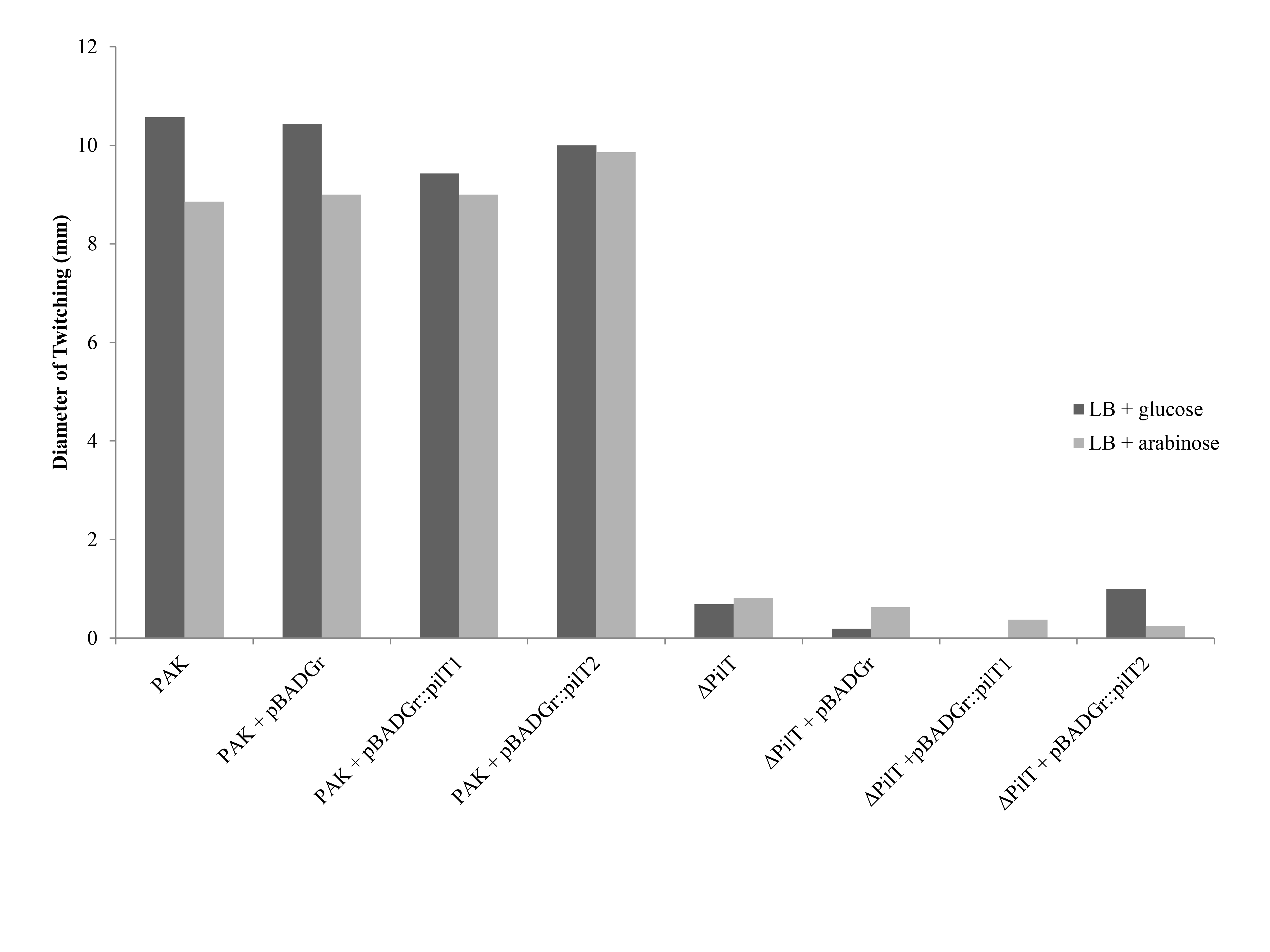

Supplement: Figure S5 — Subsurface twitching motility assay. P. aeruginosa PAK and a PilT mutant expressing PilT1 or PilT2 from B. bacteriovorus were used to assess twitching motility. PilT proteins were expressed on an arabinose inducible plasmid (pBADGr) and the P. aeruginosa cultures plated on LB agar containing 0.1% arabinose. As a control, 0.2% glucose was added to repress expression. The zone of motility was visualized using crystal violet and the diameter of each zone measured. (TIF) [file pone.0113404.s005.tif]
